# The Efficacy of Intratissue Percutaneous Electrolysis (EPI^®^) and Nutritional Factors for the Treatment of Induced Tendinopathy in Wistar Rats: Hepatic Intermediary Metabolism Effects

**DOI:** 10.3390/ijms25137315

**Published:** 2024-07-03

**Authors:** Marta Ramos-Barbero, Amalia Pérez-Jiménez, Sergio Serrano-Carmona, Khalida Mokhtari, José Antonio Lupiáñez, Eva E. Rufino-Palomares

**Affiliations:** 1 Department of Biochemistry and Molecular Biology I, Faculty of Science, University of Granada, 18071 Granada, Spain; ramosb@ugr.es (M.R.-B.); khalidafadoua1@gmail.com (K.M.); jlcara@ugr.es (J.A.L.); 2 Department of Zoology, Faculty of Science, University of Granada, 18071 Granada, Spain; 3 Sergio Serrano Fisiomedicina Avanzada, Physiotherapy Clinic, 41011 Sevilla, Spain; drserranocarmona@gmail.com

**Keywords:** Achilles tendon, aspartate, intratissue percutaneous electrolysis (EPI^®^), glycine, hydroxytyrosol, maslinic acid, nutritional factors, rehabilitation, tendinitis

## Abstract

Achilles tendinopathy (TP) is characterized as the third most common disease of the musculoskeletal system, and occurs in three phases. There is currently no evidence of effective treatment for this medical condition. In this study, the modulatory effects of the minimally invasive technique intratissue percutaneous electrolysis (EPI) and combinations of EPI with four nutritional factors included in the diet, hydroxytyrosol (HT), maslinic acid (MA), glycine, and aspartate (AA), on hepatic intermediary metabolism was examined in Wistar rats with induced tendinopathy at various stages of TP. Results obtained showed that induced tendinopathy produced alterations in the liver intermediary metabolisms of the rats. Regarding carbohydrate metabolism, a reduction in the activity of pro-inflammatory enzymes in the later stages of TP was observed following treatment with EPI alone. Among the combined treatments using nutritional factors with EPI, HT+EPI and AA+EPI had the greatest effect on reducing inflammation in the late stages of TP. In terms of lipid metabolism, the HT+EPI and AA+EPI groups showed a decrease in lipogenesis. In protein metabolism, the HT+EPI group more effectively reduced the inflammatory effects of induced TP. Treatment with EPI combined with nutritional factors might help regulate intermediary metabolism in TP disease and reduce the inflammation process.

## 1. Introduction

Achilles tendinopathy (TP) is characterized as the third most common disease of the musculoskeletal system. This disease includes changes in cellularity, such as either increased or decreased cellularity, degradation of the extracellular matrix, accumulation of ground substance, disorganised collagen fibers, and neurovascular growth [1]. This disease is included among what are known as tendinopathies, representing a clinical syndrome characterized by a combination of pain, inflammation, and impaired tendon performance, usually due to overuse, traumatism, or pathologies [2,3].

Achilles TP is a complex primary syndrome that occurs in three phases. The first phase is the inflammatory phase, with an increase in inflammatory cells in the area, especially monocytes and macrophages, to eliminate the necrotic tissue. In addition, haematoma is caused by the injury itself and platelet activation, emitting vasoactive and chemotactic agents [4]. The second phase, or proliferation phase, is characterized by the production of collagen by fibroblasts, and an increase in fibers due to the assembly of collagen molecules that form immature fibrils, which subsequently grow linearly by joining together at the ends. Finally, these fibrils grow laterally to form mature fibers that give mechanical strength and elasticity to the tendon [5]. The third and final phase is called maturation or remodelling, and its main characteristic is the increase in collagen fibers, creating an increasingly strong tissue structure [4].

In order to treat tendinopathy, diagnosis is crucial. For this, although in recent years research has been carried out into tools to aid in better and easier diagnosis of tendinopathies, ultrasound is a reliable, non-invasive, and cost-effective imaging tool to aid in the clinical diagnosis. However, further research is needed on how ultrasound techniques can be integrated with specific treatments to accurately localize treatment to the affected tendon area [6].

There is currently no evidence of effective treatment for this medical condition. The multiple treatments used for this type of injury can be divided into three main groups: invasive techniques, non-invasive techniques, and minimally invasive techniques. In general, there is little consensus on the treatment to be carried out depending on the type of tendinopathy the patient suffers from and the stage of the disease [7]. Regarding invasive techniques, surgical therapeutic approaches are used in cases in which the damage cannot be reverse by other therapies [8].

In the case of non-invasive treatments, the most common used are physiotherapy, pharmacology, eccentric movements, or isometric exercises for physical rehabilitation, to progressively increase the loading force on the tendon [9,10]. However, in recent years, there has been a trend towards the development of non-invasive therapies ranging from extracorporeal shock waves to pulsed magnetic fields. Moreover, among innovative non-invasive therapies, the addition of nutritional factors with anti-inflammatory properties could provide better and earlier recovery [3]. Among these nutritional factors can be found hydroxytyrosol (HT), maslinic acid (MA), and amino acids such as glycine and aspartic acid (AA: Gly + Asp).

Hydroxytyrosol (HT, 3,4-dihydroxyphenylethanol), one of the most potent antioxidants presented in olives, has been found to be therapeutically useful in a wide range of diseases due to its antioxidant and anti-inflammatory capacity [11,12,13]. In addition, maslinic acid (MA), 2-α, 3-β-dihydroxyolean-12-en-28-oic acid, is a triterpenoid derived from plants, such as olive, that prevents the generation of proinflammatory cytokines and oxidative stress [14,15,16], in addition to showing anticarcinogenic, antiviral, and immune system activator properties [17] and modulating glycogen metabolism [18]. Furthermore, among the amino acids (AA: Gly + Asp), glycine plays a crucial role in collagen synthesis and acts as a regulator in the systemic inflammation cascade via inhibiting TNF-α and IL-1β. Earlier research has shown that a diet rich in glycine can have positive effects on the remodeling of inflamed tendons following tendinopathy [19]. On the other hand, aspartic acid, an anaplerotic amino acid, is important for its involvement in matrix synthesis and degradation. A diet supplemented with aspartic acid leads to a reduction in liver inflammation mediated by a reduction in IL-1β and a reduction in inflammasome activity [20].

Regarding the use of minimally invasive therapies, intratissue percutaneous electrolysis (EPI) is based on the use of ultrasound applied directly to the damaged tendon using a needle that delivers microtrauma with a controlled non-thermal electrochemical ablation. This process accelerates the appearance of localized inflammation that subsequently regenerates in less time [21]. At the molecular level, the sodium hydroxide molecules alter the pH in the treated tendon, also increasing oxygen, leading to cell phagocytosis that, in turn, results in earlier and more effective repair of the injury [22]. One of the advantages of this technique is that it is minimally invasive, and the patient does not require further rehabilitation or suspension of daily activities. However, the disadvantages are that the use of EPI depends on the type of anatomical alteration and that symptomatology may persist due to nerve irritation [23]. This is why some authors believe that re-injuring the damaged area can cause a relapse, and that collagen reorganization can be achieved with eccentric work, which is non-invasive [24]. The use of EPI and eccentric exercise is becoming a common combination in the treatment of tendinopathy in athletes, since EPI is able to act on the biology of the tendon, while eccentric exercise acts on the biomechanics, facilitating the remodelling and maturation of collagen, as well as participating in neuromuscular changes that result in a decrease in the tension produced in the tendon [25]. Some authors have reported an improvement in knee function in cases of patellar tendinopathy after the use of combined EPI and eccentric exercises, with no long-term relapses [22]. Recent studies have shown that percutaneous electrolysis as a combined treatment improves the functionality of the damaged tendon, as well as decreasing post-injury pain after evaluation, following treatment ranging from 5 to 8 weeks [26]. However, to date there has been no evidence of effective injury improvement with the exclusive use of EPI [7].

The use of minimally invasive therapies such as EPI as a substitute for, or in combination with, current therapies could represent a new line of research. For this, the effect of nutrients on cell and tissue biology must be taken into account [27]. The use of natural nutritional factors with anti-inflammatory capacity, producing analgesic effects and participating in collagen metabolic pathways, such as HT, MA, or AA: Gly + Asp, could become not only a tendon-regenerative treatment, but also a preventive therapy for tendinopathies [3].

The objective of this study was to investigate the potential regulatory effects of minimally invasive EPI therapy, both alone and in combination with three potential nutraceutical compounds—specifically, hydroxytyrosol (HT), maslinic acid (MA), and a mixture of glycine and aspartate (AA)—on the activity of key enzymes involved in intermediary metabolism in the livers of Wistar rats with induced TP across various stages of the disease. For this purpose, glucose, lipid, and protein metabolisms were studied as molecular markers, using the kinetic behaviour of the main enzymes involved in the carbohydrate, lipid, and amino acid metabolisms, such as total HK (hexokinase + glucokinase), pyruvate kinase (PK), fructose 1,6-bisphosphatase (FBPase), lactate dehydrogenase (LDH), citrate synthase (CS), glucose 6-phosphate dehydrogenase (G6PDH), malic enzyme (ME), β-hydroxyacyl-CoA dehydrogenase (HOAD), fatty acid synthase (FAS), glutamate dehydrogenase (GDH), aspartate aminotransferase (AST), and alanine aminotransferase (ALT), during the development of the different stages of TP (I, I–II, II, and III).

## 2. Results

### 2.1. Growth Trial

No changes in growth performance and feed intake were observed among the different experimental groups (Table 1).

### 2.2. Enzyme Activity Related to Intermediary Metabolism

The activity of key enzymes involved in intermediary metabolism was altered in response to various treatments tested and across different phases of tendinopathy. Results for each enzyme during each phase of TP are detailed in Table 2 (Phase I), Table 3 (Phases I–II), Table 4 (Phase II), and Table 5 (Phase III).

Regarding carbohydrate metabolism, the CS enzyme did not show significant differences between treatments in Phase I and Phase III (Table 2 and Table 5). The activity of the CS enzyme was significantly reduced in groups combining EPI with nutritional factors (HT+EPI, MA+EPI, AA+EPI) compared with the DC group, but not compared with the C group (Table 3). Furthermore, during Phase II of the disease, CS activity levels were markedly lower in the EPI, HT+EPI, and AA+EPI groups compared with both the C and DC groups (Table 4). Although CS activity levels in the MA+EPI group were also lower than in the C and DC groups, these differences were not significant. With regard to the variation of CS enzyme activity in the different phases according to the treatment, significant differences were observed in the groups EPI, HT+EPI and AA+EPI, in which there were decreases in this activity in the last two phases compared with Phase I–II (Table 3, Table 4 and Table 5). The HT+EPI group showed significantly lower levels of CS activity in Phase II compared with the previous phase. Conversely, in rats treated with amino acids and EPI, there was a trend of increasing CS enzyme activity as the disease progressed; it was higher in Phase III of TP.

G6PDH enzyme activity did not exhibit significant variations between the treatments in Phase I, Phase I–II, and Phase III (Table 2, Table 3 and Table 5). However, significant differences among experimental conditions were observed within Phase II, where the AA+EPI group showed a decrease of activity compared with the healthy and diseased control groups (C and DC, Table 4). With regard to the differences between TP phases in each of the G6PDH activity treatments, it was shown that the C group had significantly lower levels in Phase III of the disease compared with the other phases (Table 2, Table 3, Table 4 and Table 5). The DC group showed significantly reduced enzyme activity in Phases I–II and III (Table 3 and Table 5). In contrast, the MA+EPI and AA+EPI groups displayed decreased activity in Phase II (Table 4).

LDH enzyme activity was not altered between treatments within the first two phases (Table 2 and Table 4). In Phase I–II, significantly lower LDH levels were observed in the groups receiving combined treatment with EPI and nutritional factors (HT+EPI, MA+EPI, AA+EPI) compared with the disease group (Table 3). In Phase III, during the remodeling phase, the EPI-treated group exhibited a significant decrement in LDH activity compared with the DC group (Table 5). When comparing enzyme activity differences between phases within the same treatment groups, a notable decrease in LDH activity was showed in Phase II (the proliferation phase) in both the C and DC groups (Table 4). The HT+EPI group exhibited decreased LDH activity in Phases I–II, followed by a notable increase in LDH activity during the later phases (Table 3, Table 4 and Table 5). In the MA+EPI and AA+EPI groups, LDH activity specifically increased during the last phase (Phase III, Table 5).

FBPase enzyme activity exhibited no notable disparities between the experimental conditions during the two first phases (Table 2 and Table 3). However, in Phase II, there was a notable decrease in FBPase activity in the EPI, HT+EPI groups, as well as in the AA+EPI group compared with the DC group, though not compared with the C group (Table 4). Moving into Phase III, it was noted that the EPI and AA+EPI groups displayed lower FBPase enzyme activity than the HT+EPI group but did not differ notably from the DC group (Table 5).

Furthermore, significant differences were observed between phases when comparing the disease progression among treatments. Specifically, the rats treated with EPI exhibited decreased FBPase activity in Phases II and III of TP (Table 4 and Table 5). Conversely, the HT+EPI and MA+EPI groups showed an increase in activity during Phase III (Table 5). Interestingly, the rats treated with AA+EPI showed decreased enzyme activity in Phase II compared with Phases I–II and III (Table 3, Table 4 and Table 5).

T-HK enzyme activity showed significant variations between experimental conditions within Phases I–II, II, and III (Table 3, Table 4 and Table 5). During the interphase (Phase I–II), a notable reduction in enzyme activity was evident across all treated groups (EPI, HT+EPI, MA+EPI, and AA+EPI) compared with group C, but not compared with DC (Table 4). In Phase II, a notable elevation in T-HK activity was observed in the EPI and MA+EPI groups in comparison to both the C and DC groups (Table 4). In the last phase, the remodelling phase, the comparatively highest activity was observed in the HT+EPI group, and the lowest activity in the AA+EPI group, with no significant difference from the control groups (C and DC, Table 5). Regarding the differences in activity between phases within the same treatment, it was observed that the EPI group demonstrated increased activity in the proliferation phase of TP (Table 4). The HT+EPI group had significantly higher T-HK activity in Phase III (Table 5).

The enzyme PK exhibited no notable differences in activity between treatments in the course of the first two phases (Table 2 and Table 3). In Phase II, PK activity was notably lower in the HT+EPI and AA+EPI groups compared with the DC group, with only HT+EPI showing a significant decrease compared with the C group (Table 4). In Phase III of TP, low levels of PK activity were observed in the groups treated with EPI and the nutritional factors in combination (MA+EPI, AA+EPI) compared with the DC group, with those in the AA+EPI group also being significantly lower than the C group (Table 5). With respect to the differences in PK enzyme activity between phases with the same treatment, a notable decline in levels was observed in the DC group in Phase I–II, increasing in Phase II and reaching a maximum in the final phase (Table 3, Table 4 and Table 5). In the HT+EPI group, there was an increase in PK activity in the last phase (Table 5). In the rats treated with MA+EPI, there was a peak of maximum activity in second phase (Table 4). Finally, the AA+EPI group exhibited elevated levels of activity in Phases I–II and III (*p* > 0.05, Table 3 and Table 5).

Regarding lipid metabolism, the activity of the ME did not result in notable differences among treatments in Phases I and I–II (Table 2 and Table 3). In the proliferation phase (Phase II), the AA+EPI groups demonstrated a reduction in enzyme activity levels compared with the DC group (Table 4). In the final phase, the HT+EPI group displayed higher levels of ME activity compared with the C group although not compared with the DC group (Table 5). On the other hand, when examining differences among stages with the same treatment, the highest ME activity in the C group was observed in Phase I–II, followed by minimal activity in Phase II (Table 3 and Table 4). The HT+EPI group exhibited an increase in ME enzyme activity in Phase III of TP (Table 5).

The FAS enzyme, on the other hand, showed changes in the later phases of the disease (*p* > 0.05, Table 3, Table 4 and Table 5). In the interphase of TP, the HT+EPI and AA+EPI groups showed significantly diminished levels of FAS enzyme function compared with the DC group (Table 3). In the second phase, all induced TP groups (DC, EPI, HT+EPI, MA+EPI and AA+EPI) obtained higher function than the healthy rats (C, Table 4). In the remodelling phase, only the EPI treated group showed lower FAS activity than the DC group (Table 5). Analyzing the differences between phases within the same treatment, it was notable that, in the C group, the highest increase in enzyme function occurred in Phase II (Table 4). In the HT+EPI and AA+EPI groups, the hello FAS activity was observed in Phase III (Table 5).

No discernible disparities were indicated for the HOAD enzyme between experimental conditions within Phases I and III (Table 2 and Table 5). In Phase I–II, the EPI group exhibited the lowest levels of function, while the rats treated with AA+EPI showed the highest levels. However, none of them showed significant differences in comparison to the healthy and disease rats (Table 3). In Phase II, the HOAD enzymes was noticeably diminished with EPI treatment compared with the C and DC groups, and the HT+EPI and AA+ EPI groups showed significantly lower HOAD activity than the C group (Table 4). On the other hand, when analyzing the differences between phases within the same treatment group, it was observed that HOAD activity in the C group exhibited an upward trend in Phase II (Table 4). In the AA+EPI group, the HOAD activity decreased significantly in Phase III (Table 3 and Table 5).

Finally, when analyzing the enzymes associated with protein metabolism, compared within the same TP phase, notable differences in GDH activity were observed in Phases I–II and II among the treatments (Table 3 and Table 4). In Phase I–II, in comparison, the EPI group exhibited notably lower GDH levels than the DC group (Table 3). In Phase II, the proliferation phase, the EPI, HT+EPI, and AA+EPI groups demonstrated decreased enzyme function compared with the C group, but not compared with the disease group (Table 4). Regarding the differences between phases within the same treatment groups, it was observed that the C group had a peak of function in Phase II (Table 4). Furthermore, in the AA+EPI group, GDH activity increased in Phase I–II and reduced in Phase II, and then was elevated, non-significantly, in Phase III (Table 3, Table 4 and Table 5).

The AST enzyme exhibited variations among the experimental conditions within Phases I–II, II, and III (Table 3, Table 4 and Table 5). In Phase I–II, the HT+EPI group demonstrated markedly reduced enzyme function levels compared with the C and DC groups (Table 3). In the second phase, the EPI, HT+EPI, and AA+EPI groups displayed decreased amounts compared with the healthy rats, but no treatment showed significant differences with respect to the diseased rats (*p* > 0.05, Table 4). In Phase III, minimal function was exhibited in the EPI group compared with the disease control group (Table 5). If focusing on the differences between phases within the same treatment, differences were only observed in the HT+EPI group, where there was a maximum AST enzyme activity in Phase III (Table 5).

ALT enzyme activity was not altered between treatments within the last two phases of the disease. In the proliferation phase, the HT+EPI group showed significantly reduced levels of function than compared with the diseased control group and the MA+EPI group showed the highest level of ALT activity, which was notably higher than the C group, although not compared with the DC group (Table 4). In Phase III, the MA+EPI group again showed the highest levels of enzyme activity, being significantly higher than the C and DC groups (Table 5). Finally, this enzyme showed differences between phases within the HT+EPI treatment, where lower levels were observed in Phase II of TP (*p* > 0.05, Table 4).

## 3. Discussion

Tendinopathies (TPs) are a multifaceted and complex group of pathologies that are characterized by common symptoms such as pain, decreased tendon function, and reduced exercise tolerance [28]. Patients with TP often receive treatments consisting of exercise and loading, different physiotherapeutic modalities, and surgical interventions, all with ambiguous efficacy [8,28]. Many of the described and commonly used therapeutic options are far from scientifically based [29]. The aim of the current research was to study the effectiveness of the EPI technique combined with the use of nutritional factors (HT, MA, and AA), to determine the effect on main pathways of intermediary metabolism that could contribute to reducing tissue inflammation, to improve the treatment of this disease in athletes. In such a way, treatment of tendinopathy with EPI and nutritional factors could represent a possible therapy to alleviate pain, promote healing, shorten the period of injury, and prevent future injuries.

The effectiveness of EPI has been demonstrated in different diseases when used in isolation [7]. Combining this technique with eccentric training has been demonstrated as one of the most effective treatments for pain improvement to date [30]. Scientific evidence has demonstrated how nutrition affects human health, making nutritional factors and components an ally in preventing diseases of all kinds [2]. HT and MA compounds have demonstrated anti-inflammatory effects in various diseases [11,13,14]. In a previous study, these anti-inflammatory compounds and a mix of Gly and Asp aminoacids (AA) demonstrated anti-inflammatory capacity in the treatment of TP [3]. In the present study, the effectiveness of EPI was shown in combination with nutritional factors (MA, HT, and AA), indicating that it could become an effective and non-invasive treatment for TP.

Certain animal and human studies have documented marked enhancements in tendon growth or collagen synthesis following diverse nutritional interventions. These include proteins and amino acids (like glycine, lysine, leucine, arginine, and glutamine), as well as vitamins C and D, alongside minerals such as manganese, copper, zinc, and phytochemicals [31,32,33]. Because the main protein in tendons is collagen, dietary treatments can be used to increase its synthesis, and, in addition, the synthesis of other proteins that aid tendon regeneration [34]. Thus, glucose metabolism is altered during tendon injury and subsequent recovery. These changes vary widely and include elevations in glucose, lactate, and pyruvate levels in healing human Achilles tendons [31] and stimulation of glycolysis, lactate synthesis, and pyruvate synthesis [27]. Similarly, to these authors, the results obtained in this research from the group with induced tendinopathy showed a clear tendency to increase glucose metabolism, which did not occur in the healthy group without induced tendinopathy. In our results, we also observed an alteration in carbohydrate metabolism in response to TP induced in the livers of rats. Treatment of TP using minimally invasive techniques such as EPI and nutritional factors could modulate this altered metabolism in order to supply required metabolites or energy to face each of the phases of this pathology.

According to our results, treatment based on the use of the EPI technique produced a decrease in several enzymes involved in carbohydrate metabolism. Firstly, the enzyme CS, which is involved in the metabolization of glucose to generate ATP via the glycolysis, tricarboxylic acid, and electron transport chain pathways [35], was affected with reduced activity in Phase II. This was probably due to the action of EPI in the earlier stages of the disease as it is a shock therapy, where there is an increase in inflammation in the first phase, which accelerates the regenerative process in the earlier stages, and therefore, there is a lower energy requirement in the following phases of the disease [22]. On the other hand, the role of FBPase is to contribute to gluconeogenesis, a process that can be affected by inflammation, thereby contributing to metabolic stress [36]. Therefore, the effect of EPI in the early stages of the disease, where there was increased inflammation, provided elevated levels of this enzyme, with FBPase activity being reduced in later stages due to the effectiveness of the treatment in reducing inflammatory processes in a shorter time than without EPI treatment [36]. Moreover, in this second phase of the disease, the proliferation phase, the groups in this study treated with nutritional factors and EPI, HT+EPI, and AA+EPI produced the same results for CS and FBPase enzymes, indicating that this decrease in activity may have been mainly due to the action of EPI. However, it should be noted that the groups combining nutritional factors with EPI showed significantly lower levels than the group treated with EPI alone; thus, HT and AA contributed to a reduction in inflammation. So, HT can modulate several key inflammation-related processes by decreasing enzymes involved in these processes [37]. Glycine can decrease the inflammation produced by TP, via inhibiting pro-inflammatory proteins [38]. The involvement of these nutritional factors in reducing the activity of these inflammatory enzymes has been demonstrated in previous studies [3].

Additionally, our results for EPI and EPI + nutritional factors (HT+EPI, MA+EPI and AA+EPI) showed a reduction of PK activity in the late phases of the disease. This fact could indicate an early anti-inflammatory effect, as PK regulates glycolysis and is influenced by the metabolic effects of HT, MA. and AA. When comparing the percentage reduction of PK activity from Phase I–II to Phase III of TP with the results of previous studies, we observed that the groups treated with EPI, either alone or in combination with nutritional factors, exhibited lower activity compared with the groups treated solely with nutritional factors, 18% vs. 3% reduction in those phases, respectively [3]. Focusing on the results in the present study for in each of the separate phases, PK activity was higher in Phases I–II in the previous research (28% increase compared with the DC group) [3] than in the present study (10% decrease compared with the DC group). This suggests an acceleration in tissue damage regeneration process, when EPI is applied. So, PK reduction began in Phase I–II in the present study vs. Phase II in previous studies in which EPI was not used.

On the other hand, when we determine the potential synergistic effects between EPI and nutritional factors, only HT demonstrated this. So, these results indicated that the efficacy of the combined treatment was more effective. In this sense, HT have contributed through its anti-inflammatory and antioxidant effects [16].

Continuing with carbohydrate metabolism, the LDH enzyme showed reduced activity in the three combined treatment groups, HT+EPI, MA+EPI, and AA+EPI, from the early stages of TP and in the EPI group in the late stages. Earlier research demonstrated that severe inflammation can trigger elevated LDH production, linked to an increased release of proinflammatory cytokines [39,40] and therefore, its decrease implies a reduction of inflammation leading to effective tendon repair.

In general, both EPI and the combined treatment with nutritional factors and EPI reversed the changes produced by TP in the hepatic carbohydrate metabolism.

Among the results of the enzyme activities for lipid metabolism, FAS activity was found to be reduced in the HT+EPI and AA+EPI groups in Phase I–II, and in the EPI group in Phase III. This may be due to the fact that the FAS enzyme participates in various metabolic processes such as membrane synthesis, proliferation, protein modification, and energy redistribution [41,42]. Therefore, its reduction indicated early recovery of the tendon and that damage repair mechanisms were no longer needed, coinciding with previous results [3]. These data reinforce the idea of the greater efficacy of these two combined treatments, HT+EPI and AA+EPI, in the recovery of Achilles TP. Likewise, this fact is coupled with the reduction of ME and HOAD activity with these two treatments. This effect could be due to amino acids breaking down glycine and aspartic acid for use in metabolic pathways of energy generation from beta-oxidation of fatty acids [43,44]. In addition, ME is involved in the regenerative processes of the tendon, this enzyme being unnecessary when the tendon has already been repaired, as it would not need to supply NADPH molecules to the liver [45].

Finally, our treatments produced effects on protein metabolism, where GDH, AST, and ALT levels were affected, as the liver is the major organ involved in this type of metabolism, deleting the amino group in protein catabolism thanks to transaminases [16]. Thus, in Phase I–II, the EPI group had lower levels of GDH due to a decrease in tissue damage and inflammation, as has been shown in other diseases [46]. In the present study, the EPI and HT+EPI groups showed a decrease in AST activity, with the combined group showing positive results earlier during the TP phases. The groups treated with EPI alone and AA alone were those with the greatest percentage of reduction in AST activity, being approximately 25% with respect to the DC group. It should be noted that there was synergy between HT and EPI, which translated into a greater reduction in AST activity when these treatments were combined. However, this synergy presented a reduction in AST activity of approximately 17%, which was lower than the percentage presented following EPI treatment alone [3]. An increase in this enzyme has been observed in inflammatory diseases and diseases affecting tissue, implying decreased early tendon recovery [46]. As can be seen, the anti-inflammatory effects of HT added to the rapid action of the EPI technique for better repair [47].

Additionally, in the early phases, the HT+EPI group showed a decrease in ALT activity, which could be attributed to the intense activity observed in the later phases of TP. During these stages, mature tendon fibers are formed, allowing the tissue to regain its elasticity and strength and restore normal function [48]. The MA+EPI group presented an increase in ALT activity in the last phase; this may have been due to the fact that in the Phases II and III of TP there is vigorous activity leading to the formation of mature tendon fibers, facilitating the restoration of elasticity and strength essential for the tissue to regain its normal function [48]. In a previous study, an increase in ALT activity was observed in groups of rats with induced TP treated with MA alone [3]. This could indicate a greater involvement of EPI in reducing the activity of this enzyme, as it is able to act on tendon biology, activating the mechanisms of phagocytosis and tendon repair, while eccentric exercise acts on tendon biomechanics, facilitating collagen remodelling and maturation, as well as participating in neuromuscular changes that result in a decrease in the tension produced in the tendon [25]. Comparing the results obtained in the present study with previous studies, a greater reduction in ALT activity was observed in the EPI group, approximately 41%, along with a synergy between AA and EPI treatment with respect to the AA group alone, reducing activity up to 27%, and between HT and EPI treatment, producing a reduction in activity of approximately 53% [3].

**Figure 1:**
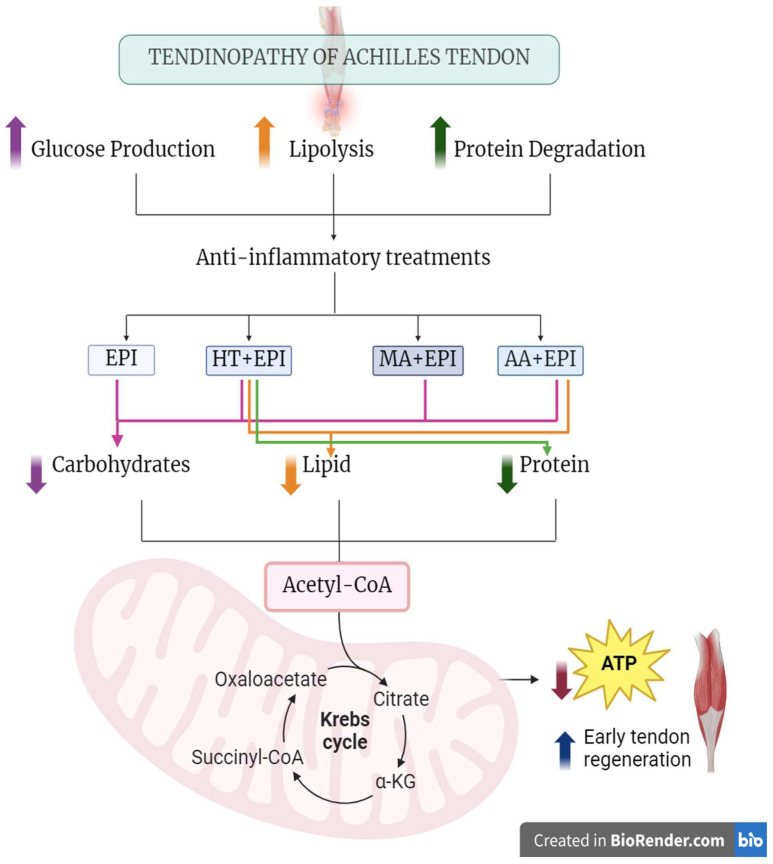
Change in hepatic metabolism produced by TP treatments.

In general terms, the change in hepatic metabolism produced by each of the TP treatments can be seen in Figure 1.

Future tendinopathy research should integrate findings from laboratory and clinical studies to better understand the disease. Advances in understanding tendinopathy’s pathogenesis will enhance cellular and molecular treatments. Future research should explore genetic and epigenetic changes, effective stem cell types, scaffold materials, and disease-modifying agents. So, combining various treatment modalities such as physical therapy, biological interventions, and pharmacological treatments, combined with healthy nutrition and lifestyle can help to achieve synergistic effects.

## 4. Materials and Methods

### 4.1. Animal Models and Experimental Conditions

In this study, the experimental conditions, ethical approval, and use of animal models were the same as those used by Ramos-Barbero et al. (2024) [3]. The rats were distributed into 6 groups (4 replicates in each): Healthy control (C), diseased control (DC), intratissue percutaneous electrolysis (EPI), intratissue percutaneous electrolysis combined with hydroxytyrosol (EPI+HT), intratissue percutaneous electrolysis combined with maslinic acid (EPI+MA), and intratissue percutaneous electrolysis combined with glycine and aspartate amino acids (EPI+AA: Gly + Asp). Proximate composition of the experimental diets were determined based on AOAC methods [49] and it is presented in Table 6.

The research had a total duration of 41 days. On day 1, Achilles TP was initiated in the right Achilles tendon through the administration of 50 µg of collagenase type I per 300 g of body weight (Sigma-Aldrich, St. Louis, MO, USA). This induction was performed in all groups except the control group. In the control group, saline solution was used instead of collagenase. On day 6, matching with the inflammatory phase, EPI treatment was administered via a galvanic current applied to the injured tendon using an acupuncture needle (0.25 × 25 mm) as a cathodic flow electrode, using a medically certified device (Directive 93/42/EEC) (EPI Advanced Medicine, Barcelona, Spain). Following a medial approach, the needle was directed to its intended target (the lesion focus) using ultrasound. An average current of 560.25 mJ was delivered, with an intensity of 3 mA for 4 s. The mean EPI dose for each of the groups adjusted for the individual animals’ weight was: 642.67 ± 25.23 mJ for EPI, 495.42 ± 16.25 mJ for HT+EPI, 555.83 ± 14.70 mJ for MA + EPI, and 547.08 ± 10.81 mJ for AA + EPI.

Sampling was carried out on day 6 (inflammatory phase) for only the C and DC treatments. On days 13, 26, and 40 (phases of TP), samples from all the experimental groups were collected. The rats were anesthetized under the same conditions used by Ramos-Barbero et al. (2024) [3]. Subsequently, 3-5 mL of blood was withdrawn from the heart with the help of a needle applied in the left costal area of the rat. The animals were then sacrificed with an overdose of sodium pentobarbital and the liver was excised and promptly frozen in liquid nitrogen for subsequent biochemical analysis. The extracted blood was centrifuged, the liver was homogenized, and all samples were aliquoted and stored at −80 °C.

### 4.2. Analysis of Enzymes Involved in Intermediary Metabolism

The specific enzyme activities of total HK (hexokinase + glucokinase), pyruvate kinase (PK), fructose 1,6-bisphosphatase (FBPase), lactate dehydrogenase (LDH), citrate synthase (CS), glucose 6-phosphate dehydrogenase (G6PDH), malic enzyme (ME), β-hydroxyacyl-CoA dehydrogenase (HOAD), fatty acid synthase (FAS), glutamate dehydrogenase (GDH), aspartate aminotransferase (AST), and alanine aminotransferase (ALT), were determined following the methods described previously [3,50,51]. The measurement of enzyme activity was performed through determining the variation of the optical density (OD) at 37 °C in a Synergy HTX plate reader using Gen 5™ software (version 2.0.0).

Each enzyme’s activity was quantified in milliunits per milligram of soluble protein, aligning with its specific function. One unit of enzyme activity was defined as the quantity of enzyme needed to convert 1 µmol of substrate per minute under the specified test conditions. Finally, the Bradford method [52] was used to measure the quantity of soluble proteins, using bovine serum albumin as a benchmark.

### 4.3. Statistical Analysis

The results are presented as the mean ± standard error of the mean (SEM). The effects of the various treatments on all the parameters studied were analyzed using two-way analysis of variance (two-way ANOVA), followed by Tukey’s HSD test. Given the observed interaction between the analyzed factors in all parameters assessed, one-way ANOVA was performed for each factor independently, followed by Tukey’s HSD test. A confidence interval of 95% was used, and the differences were considered significant at values of *p* < 0.05. All statistical analyses including those performed to confirm the adequate sample size and statistical power of the present study were carried out using the IBM SPSS Statistics program software (IBM^®^, Armonk, NY, USA) (version 25).

## 5. Conclusions

Induced tendinopathy leads to alterations in liver metabolism. In this context, we can highlight the following points:

– In carbohydrate metabolism, a reduction in the activity of pro-inflammatory enzymes in the later stages of TP was observed following treatment with EPI alone. All combined treatments of nutritional factors with EPI were shown to reduce pro-inflammatory enzymes in this metabolism. However, HT+EPI and AA+EPI had the greatest effect on reducing inflammation in the late stages of TP, and this efficacy was even greater than in the group treated with EPI alone.
– In terms of lipid metabolism, the HT+EPI and AA+EPI groups showed a decrease in lipogenesis.
– In terms of protein metabolism, the HT+EPI group was more effective.

From the above, it can be concluded that treatment with EPI combined with nutritional supplements, especially HT, could help to regulate the intermediary metabolism in TP and reduce the inflammatory process, which could be an alternative to the current invasive and ineffective treatment of tendinopathies.

## Figures and Tables

**Table 1:** Growth performance and feed intake of different experimental groups: C (healthy control), DC (diseased control), EPI (intratissue percutaneous electrolysis), HT+EPI (hydroxytyrosol and intratissue percutaneous electrolysis), MA+EPI (maslinic acid and intratissue percutaneous electrolysis), AA+EPI (Gly + Asp) (amino acids glycine and aspartate and intratissue percutaneous electrolysis).

Experimental Groups	Initial Weight (g)	Final Weight (g)	Weight Gain (g/Kg/Day)	Feed Intake (g/Kg/Day)
C	316.8 ± 3.3	470.0 ± 24.4	11.8 ± 0.8	73.8 ± 1.2
DC	317.7 ± 3.3	483.0 ± 7.2	10.8 ± 0.9	72.84 ± 0.9
EPI	314.4 ± 5.4	453.8 ± 12.1	11.5 ± 0.4	74.3 ± 1.1
EPI+HT	323.5 ± 4.0	492.0 ± 39.8	10.2 ± 0.5	72.1 ± 0.9
EPI+MA	324.4 ± 3.9	491.0 ± 3.7	11.5 ± 0.7	72.5 ± 2.6
EPI+AA	314.2 ± 5.6	480.0 ± 7.0	9.5 ± 0.5	73.9 ± 1.3

Values are expressed as mean ± SEM (*n* = 4) and were deemed statistically significant at *p* < 0.05.

**Table 2:** Phase I. The impact of various nutritional treatments and stages of tendinopathy on the activity (nmol/min/mg protein) of enzymes citrate synthase (CS), glucose-6-phosphate dehydrogenase (G6PDH), lactate dehydrogenase (LDH), fructose bisphosphatase (FBPase), total hexokinase (T-HK), pyruvate kinase (PK), malic enzyme (ME), fatty acid synthase (FAS), hydroxyacyl-CoA dehydrogenase (HOAD), glutamate dehydrogenase (GDH), aspartate aminotransferase (AST), and alanine aminotransferase (ALT) in rat liver samples with and without induced tendinopathy. C denotes healthy control and DC denotes diseased control.

Treatments	Control (C)	Diseased Control (DC)
Enzymes of carbohydrate metabolism		
CS	6.41 ± 0.59	8.08 ± 0.63
G6PDH	27.49 ± 2.37 ^B^	32.53 ± 2.68 ^B^
LDH	3417.5 ± 92.5 ^B^	3366.6 ± 197.4 ^AB^
FBPase	34.42 ± 1.42	40.87 ± 1.88
T-HK	1.69 ± 0.13 ^B^	1.93 ± 0.48
PK	229.07 ± 10.43	317.32 ± 13.48 ^B^
Enzymes of lipid metabolism		
ME	4.86 ± 0.45 ^AB^	7.05 ± 0.77
FAS	0.81 ± 0.02 ^A^	1.12 ± 0.05
HOAD	145.03 ± 10.84 ^A^	171.03 ± 12.71
Enzymes of amino acid metabolism		
GDH	530.07 ± 16.73 ^A^	645.38 ± 85.49
AST	702.8 ± 51.85	743.12 ± 79.36
ALT	91.16 ± 12.54	132.21 ± 4.81

Values are expressed as mean ± SEM (*n* = 4) and deemed statistically significant at *p* < 0.05. Uppercase letters denote significant differences between stages of tendinopathy within each experimental treatment.

**Table 3:** Phase I–II. The impact of various nutritional treatments and stages of tendinopathy on the activity (nmol/min/mg protein) of hepatic metabolism key enzymes in Wistar rats. C denotes healthy control and DC denotes diseased control. EPI (intratissue percutaneous electrolysis), HT+EPI (hydroxytyrosol and intratissue percutaneous electrolysis), MA+EPI (maslinic acid and intratissue percutaneous electrolysis), AA+EPI (Gly + Asp) (amino acids glycine and aspartate and intratissue percutaneous electrolysis).

Treatment	C	DC	EPI	HT+EPI	MA+EPI	AA+EPI
Enzymes of carbohydrate metabolism						
CS	5.70 ± 0.51 ^a^	9.58 ± 1.23 ^b^	9.04 ± 0.34 ^Bb^	5.19 ± 0.16 ^Ba^	5.40 ± 0.62 ^a^	3.33 ± 0.08 ^Aa^
G6PDH	26.68 ± 2.52 ^B^	21.37 ± 1.9 ^A^	26.89 ± 4.58	15.12 ± 1.8	26.12 ± 4.59 ^AB^	24.44 ± 2.12 ^B^
LDH	3290 ± 184.2 ^Bab^	4079.4 ± 247 ^Bb^	3235.5 ± 126.5 ^ab^	2595 ± 166.9 ^Aa^	2568.3 ± 52.4 ^Aa^	2918.5 ± 290.8 ^ABa^
FBPase	41.06 ± 5.64	39.15 ± 1.39	42.85 ± 1.30 ^B^	31.94 ± 2.98 ^A^	39.52 ± 3.51 ^A^	44.65 ± 4.85 ^B^
T-HK	2.58 ± 0.28 ^Cb^	1.80 ± 0.04 ^ab^	1.74 ± 0.15 ^Aa^	1.28 ± 0.20 ^Aa^	1.25 ± 0.01 ^a^	1.40 ± 0.22 ^a^
PK	244.27 ± 38.81	241.84 ± 12.30 ^A^	250.73 ± 25.32	197.78 ± 14.19 ^A^	217.20 ± 4.09 ^A^	237.66 ± 9.94 ^B^
Enzymes of lipid metabolism						
ME	6.19 ± 0.71 ^B^	6.53 ± 0.68	5.09 ± 0.77	4.66 ± 0.44 ^A^	4.88 ± 0.35	4.51 ± 0.99
FAS	1.30 ± 0.11 ^ABab^	1.85 ± 0.16 ^b^	1.15 ± 0.09 ^ab^	0.72 ± 0.04 ^Aa^	1.61 ± 0.39 ^b^	0.49 ± 0.03 ^Aa^
HOAD	189.87 ± 19.85 ^ABab^	184.88 ± 11.19 ^ab^	129.68 ± 4.24 ^a^	192.28 ± 10.29 ^ab^	179.11 ± 18.37 ^ab^	203.18 ± 15.71 ^Bb^
Enzymes of amino acid metabolism						
GDH	683.82 ± 79.60 ^ABab^	866.43 ± 88.68 ^b^	467.52 ± 39.63 ^a^	568.79 ± 42.40 ^ab^	623.33 ± 74.10 ^ab^	820.49 ± 75.45 ^Bb^
AST	979.04 ± 116.16 ^b^	1065.56 ± 106.69 ^b^	777.38 ± 71.27 ^ab^	547.34 ± 23.07 ^Aa^	778.32 ± 99.64 ^ab^	896.10 ± 93.33 ^ab^
ALT	196.27 ± 55.93	105.34 ± 11.18	97.04 ± 22.21	130.65 ± 7.71 ^B^	130.75 ± 13.27	157.93 ± 28.09

Values are expressed as mean ± SEM (*n* = 4) and deemed statistically significant at *p* < 0.05. Lower case letters denote significant differences between treatments within each stage of the tendinopathy. Uppercase letters denote significant differences between stages of tendinopathy within each experimental treatment.

**Table 4:** Phase II. The impact of various nutritional treatments and stages of tendinopathy on the activity (nmol/min/mg protein) of hepatic metabolism key enzymes in Wistar rats. C denotes healthy control and DC denotes diseased control. EPI (intratissue percutaneous electrolysis), HT+EPI (hydroxytyrosol and intratissue percutaneous electrolysis), MA+EPI (maslinic acid and intratissue percutaneous electrolysis), AA+EPI (Gly + Asp) (amino acids glycine and aspartate and intratissue percutaneous electrolysis).

Treatment	C	DC	EPI	HT+EPI	MA+EPI	AA+EPI
Enzymes of carbohydrate metabolism						
CS	7.84 ± 0.98 ^b^	7.25 ± 0.60 ^b^	4.58 ± 0.36 ^Aa^	4.27 ± 0.33 ^Aa^	5.55 ± 0.47 ^ab^	4.02 ± 0.33 ^ABa^
G6PDH	25.57 ± 1.04 ^Bb^	26.36 ± 3.62 ^ABb^	19.38 ± 1.74 ^ab^	24.79 ± 5.8 ^b^	16.81 ± 1.26 ^Aab^	11.34 ± 1.48 ^Aa^
LDH	2307.5 ± 56.9 ^A^	2875.4 ± 397 ^A^	3440.9 ± 352.4	3540.7 ± 264.1 ^B^	3109.6 ± 198.4 ^AB^	2511.3 ± 335.5 ^A^
FBPase	32.62 ± 2.46 ^a^	43.73 ± 3.93 ^b^	29.57 ± 1.74 ^Aa^	28.28 ± 0.52 ^Aa^	44.15 ± 1.66 ^ABb^	28.66 ± 1.56 ^Aa^
T-HK	0.66 ± 0.04 ^Aa^	1.32 ± 0.11 ^b^	2.22 ± 0.03 ^Bc^	1.27 ± 0.09 ^Ab^	2.17 ± 0.14 ^c^	0.98 ± 0.09 ^ab^
PK	225.47 ± 8.62 ^bc^	268.66 ± 12.6 ^ABc^	224.20 ± 20.94 ^bc^	163.56 ± 17.5 ^Aab^	296.52 ± 23.87 ^Bc^	145.01 ± 8.25 ^Aa^
Enzymes of lipid metabolism						
ME	2.65 ± 0.03 ^Aa^	4.45 ± 0.44 ^bc^	4.80 ± 0.04 ^c^	3.23 ± 0.53 ^Aab^	5.43 ± 0.32 ^c^	2.34 ± 0.04 ^a^
FAS	2.00 ± 0.16 ^Cb^	1.01 ± 0.06 ^a^	1.15 ± 0.07 ^a^	0.92 ± 0.07 ^Aa^	1.08 ± 0.13 ^a^	0.91 ± 0.10 ^Ba^
HOAD	228.06 ± 14.03 ^Bc^	210.56 ± 17.16 ^bc^	152.20 ± 11.91 ^a^	157.45 ± 14.14 ^ab^	228.61 ± 8.75 ^c^	162.75 ± 7.62 ^ABab^
Enzymes of amino acid metabolism						
GDH	883.35 ± 69.38 ^Bb^	645.12 ± 5.95 ^a^	586.16 ± 27.45 ^a^	553.97 ± 50.8 ^a^	691.84 ± 42.54 ^ab^	556.44 ± 39.56 ^Aa^
AST	1046.51 ± 100.50 ^b^	802.49 ± 19.99 ^ab^	722.68 ± 49.84 ^a^	743.18 ± 97.81 ^Aa^	879.93 ± 56.69 ^ab^	684.03 ± 36.63 ^a^
ALT	125.10 ± 9.53 ^ab^	156.45 ± 17.84 ^bc^	113.68 ± 20.34 ^ab^	80.43 ± 10.40 ^Aa^	206.31 ± 9.47 ^c^	124.96 ± 16.91 ^ab^

Values are expressed as mean ± SEM (*n* = 4) and deemed statistically significant at *p* < 0.05. Lower case letters denote significant differences between treatments within each stage of the tendinopathy. Uppercase letters denote significant differences between stages of tendinopathy within each experimental treatment.

**Table 5:** Phase III. The impact of various nutritional treatments and stages of tendinopathy on the activity (nmol/min/mg protein) of hepatic metabolism key enzymes in Wistar rats. C denotes healthy control and DC denotes diseased control. EPI (intratissue percutaneous electrolysis), HT+EPI (hydroxytyrosol and intratissue percutaneous electrolysis), MA+EPI (maslinic acid and intratissue percutaneous electrolysis), AA+EPI (Gly + Asp) (amino acids glycine and aspartate and intratissue percutaneous electrolysis).

Treatment	C	DC	EPI	HT+EPI	MA+EPI	AA+EPI
Enzymes of carbohydrate metabolism						
CS	5.39 ± 0.48	7.39 ± 1.74	4.19 ± 0.31 ^A^	4.61 ± 0.16 ^AB^	7.36 ± 1.11	5.19 ± 0.72 ^B^
G6PDH	13.15 ± 0.54 ^A^	18.63 ± 0.93 ^A^	30.18 ± 10.46	22.68 ± 1.5	29.94 ± 0.99 ^B^	24.58 ± 3.1 ^B^
LDH	4031.8 ± 269.1 ^Bab^	4415.8 ± 245.6 ^Bb^	3107.1 ± 282.3 ^a^	4391.1 ± 105.1 ^Cb^	3750.3 ± 343 ^Bab^	4078.2 ± 275.2 ^Bab^
FBPase	41.38 ± 7.60 ^a^	50.95 ± 4.73 ^ab^	35.91 ± 1.75 ^Aa^	63.53 ± 2.75 ^Bb^	53.15 ± 2.33 ^Bab^	42.67 ± 1.81 ^Ba^
T-HK	2.26 ± 0.22 ^BCab^	1.94 ± 0.22 ^ab^	1.85 ± 0.08 ^ABab^	2.34 ± 0.09 ^Bb^	2.17 ± 0.41 ^ab^	1.28 ± 0.08 ^a^
PK	327.57 ± 40.60 ^bc^	390.44 ± 24.37 ^Cc^	283.03 ± 16.24 ^ab^	333.94 ± 20.07 ^Bbc^	235.45 ± 3.95 ^Aab^	219.75 ± 8.94 ^Ba^
Enzymes of lipid metabolism						
ME	3.97 ± 0.65 ^ABa^	5.57 ± 0.82 ^ab^	4.70 ± 0.28 ^ab^	7.45 ± 0.70 ^Bb^	6.38 ± 0.82 ^ab^	4.68 ± 0.40 ^ab^
FAS	1.44 ± 0.11 ^Bab^	2.12 ± 0.56 ^b^	1.00 ± 0.04 ^a^	1.44 ± 0.12 ^Bab^	2.00 ± 0.09 ^ab^	1.28 ± 0.04 ^Cab^
HOAD	177.78 ± 10.33 ^AB^	204.53 ± 14.98	132.37 ± 3.70	203.69 ± 18.64	170.08 ± 30.72	139.93 ± 20.94 ^A^
Enzymes of amino acid metabolism						
GDH	726.83 ± 87.22 ^AB^	792.48 ± 95.66	470.81 ± 36.44	737.33 ± 74.87	801.92 ± 94.06	621.93 ± 47.03 ^AB^
AST	973.28 ± 88.95 ^ab^	1073.27 ± 84.09 ^b^	652.28 ± 45.99 ^a^	1120.79 ± 109.4 ^Bb^	980.19 ± 71.71 ^ab^	838.41 ± 52.89 ^ab^
ALT	115.35 ± 17.22 ^a^	112.03 ± 11.65 ^a^	155.05 ± 20.95 ^ab^	164.69 ± 14.45 ^Bab^	213.74 ± 36.39 ^b^	140.48 ± 12.95 ^ab^

Values are expressed as mean ± SEM (*n* = 4) and deemed statistically significant at *p* < 0.05. Lower case letters denote significant differences between treatments within each stage of the tendinopathy. Uppercase letters denote significant differences between stages of tendinopathy within each experimental treatment.

**Table 6:** Proximate analyses (% dry matter) of diets.

Proximate Analysis	C/DC/EPI	HT+EPI	MA+EPI	AA+EPI
Moisture	9.48	8.74	8.11	8.15
Crude protein	14.58	14.62	13.96	17.48
Crude lipid	3.76	4.37	2.76	3.60
Ash	3.77	3.87	3.93	3.84
Nitrogen free extract ^1^	68.41	68.40	71.24	66.93
Energetic value (KJ/100 g dry weight)	1529	1552	1528	1547

^1^ Nitrogen free extract = 100 − (crude protein + crude lipid + ash).

## Data Availability

Data will be made available on request.
